# The impact of systemic hypertension on outcomes in hospitalized COVID-19 patients – a systematic review

**DOI:** 10.4314/ahs.v22i4.57

**Published:** 2022-12

**Authors:** James Ayodele Ogunmodede, Adebusola Jane Ogunmodede, Bolade Folasade Dele-Ojo, Idris Abiola Yusuf, Adeseye Abiodun Akintunde, Oladimeji Akeem Bolarinwa, Taiye Peter Omojasola, Ibraheem Adeola Katibi, Ayodele Babatunde Omotoso

**Affiliations:** 1 Department of Medicine, University of Ilorin, Ilorin, Kwara state, Nigeria; 2 Department of Behavioural sciences, University of Ilorin Teaching Hospital, Ilorin, Kwara state, Nigeria; 3 Department of Medicine, Ekiti state University, Ado-Ekiti, Ekiti state; 4 Department of Internal Medicine, Dalhatu Araf Specialist Hospital, Keffi, Nasarawa state Nigeria; 5 Department of Medicine, Ladoke Akintola University of Technology, Ogbomosho, Oyo state; 6 Department of Epidemiology and Community Health, University of Ilorin, Ilorin, Kwara state, Nigeria; 7 Omolola Specialist Hospital, Ilorin, Kwara state, Nigeria

**Keywords:** Hypertension and COVID-19, SARS -Cov-2, severity, mortality, ICU care, mechanical ventilation

## Abstract

**Background:**

Several observational reports from different parts of the world have shown that systemic hypertension (hypertension) was the single commonest comorbid condition in hospitalized COVID-19 patients. Hypertension is also the most prevalent comorbidity reported among patients who developed severe disease, were admitted to Intensive Care Unit, needed mechanical ventilatory support, or who died on admission. The objective of this systematic review is to study the association between hypertension and specific clinical outcomes of COVID-19 disease which are- development of severe COVID-19 disease, need for admission in the intensive care unit (ICU) or critical care unit (CCU), need for mechanical ventilation or death

**Methods:**

We searched the PubMed, SCOPUS, and Google Scholar databases up till June 28, 2020 for original research articles that documented the risk factors of mortality in patients with COVID-19 using the PRISMA guideline.

**Results:**

One hundred and eighty-two articles were identified using pre-specified search criteria, of which 33 met the study inclusion criteria. Only three were prospective studies. Most studies documented hypertension as the most prevalent comorbidity. The association of hypertension with development of severe COVID-19 disease was not conclusive, majority of studies however found an associated with mortality.

**Conclusion:**

Hypertension affects the clinical course and outcome of COVID-19 disease in many cohorts. Prospective studies are needed to further understand this relationship.

## Introduction

Starting from a cluster of acute respiratory infection cases in Wuhan, Hubei Province China in December 2019 infections with the severe acute respiratory syndrome coronavirus 2 (SARS-CoV-2), 1,2 has grown to a pandemic of monumental proportions with 21,259,147 cases and 760,840 deaths as at August 14, 2020^3^ leaving in its wake grave economic and social consequences. Initially called 2019 novel coronavirus (2019nCov) on January 3, 2020 after it was identified from thorough put sequencing of broncheoalveolar lavage fluid from a patient, the World Health Organization (WHO) designated it SARS-CoV-2 and gave the name Coronavirus disease-2019 (COVID-19) to the clinical condition caused by it on February 11, 2020^4^ and declared the disease a pandemic on March 11, 2020 as it rapidly escalated.

Early reports from Wuhan about the epidemiology of the disease indicated the high prevalence of comorbid conditions such as systemic hypertension (hypertension), diabetes mellitus, coronary artery disease, cancer and other chronic illnesses among hospitalized cases of Covid 19. Up to half of admitted patients in some early reports from Hubei province reportedly had comorbidities and this proportion increased to as high as two-thirds in those who developed severe disease requiring Intensive Care Unit (ICU) care or leading to death.^5^, ^6^ Several observational reports from different parts of the world - Wuhan, China, Chinese cities other than Wuhan, USA, Italy and Israel, have shown that systemic hypertension was the single commonest comorbid condition in hospitalized Covid-19 patients.^7^–^11^ Hypertension is also the most prevalent comorbidity reported among patients who developed severe disease, needed mechanical ventilatory support, were admitted to ICU or who died on admission.^12^–^15^An Italian database reported that up to73% of patients who have died in the pandemic had hypertension. 16, 17

This raised multiple questions regarding the impact of hypertension on the clinical course of COVID-19 disease. The objective of this systematic review is to study the association between hypertension and specific clinical outcomes of COVID-19 disease which are- development of severe COVID-19 disease, need for admission in the intensive care unit (ICU) or critical care unit (CCU), need for mechanical ventilation or death.

## Methodology

### Search Strategy

We systematically searched the PubMed, SCOPUS, and Google Scholar database up till June 28, 2020 for articles that documented the risk factors of mortality in patients with COVID-19. We used MeSH key words that included coronavirus, COVID-19, COVID-19 Mortality, systemic hypertension, cardiovascular disease and mortality. In the first round of search, hypertension was variously combined with COVID-19, Coronavirus and coronavirus 2019 while in the second round, it was variously combined with COVID-19 mortality, COVID-19 severity and COVID-19 outcomes.

We retrieved all the available literature published in English language on COVID-19 that reported patients' comorbidity profiles and the outcomes in patients with systemic hypertension. The analysis was conducted following the Preferred Reporting Items for Systematic Reviews and Meta-Analyses (PRISMA)^18^

### Eligibility Criteria

#### Inclusion criteria

This study included only full-length original research articles that were published in English language and in which patients were diagnosed for COVD- 19 based on the World Health Organization (WHO) recommendation of positive result of a reverse transcriptase-polymerase chain reaction (RT-PCR) assay of nasal and/or throat-swab specimens. Only studies of hospitalised patients were included. 19

The primary outcome studied was the prevalence of hypertension recorded in each cohort of patients with COVID-19. The secondary outcomes were the association of hypertension with i. developing severe COVID-19 disease, ii. need for admission in the intensive care unit (ICU) or critical care unit (CCU), iii. need for mechanical ventilation and iv. death.

#### Exclusion criteria

We excluded systematic or narrative review articles, meta-analyses, letters to the editor which did not report original research, case reports and small case series with less than 20 patients. Publications in languages other than English and research in paediatric patients younger than 18 years of age were also excluded.

## Results

**Figure 1:**
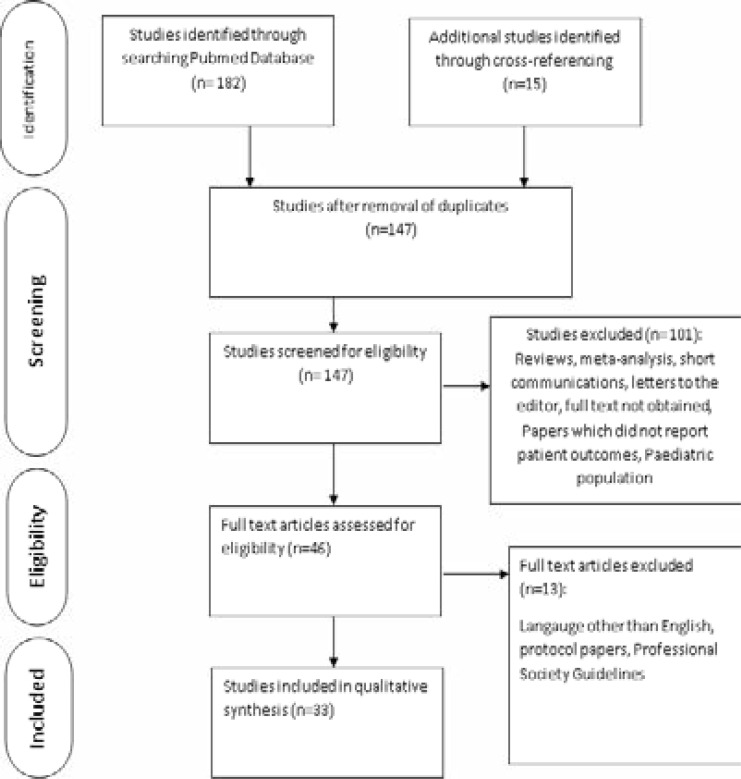
PRISMA Flow Diagram of the Study

The initial search returned 182 publications from PubMed, SCOPUS, and Google scholar. Additional 15 publications were obtained through cross-referencing. 147 publications remained after the removal of duplicates. After screening them for eligibility, 101 records were excluded. The 46 remaining articles were then evaluated for eligibility by assessing their full text. 13 full-text articles were excluded because they were written in other languages than English and some were protocol papers. Thereby, 33 studies with a total of 94,765 patients were included in the final analysis (Figure 1, Table 1)

**Table 1:** The impact of systemic hypertension on outcomes in hospitalized COVID-19 patients

	Author	Type of Study	Sample size	Country	Median age (IQR)/ Mean age + SD	% Male	Type of pt	Prevalence of Hypt	Prevalence of DM	Prevalence of other CVDs	Outcome measure	% with Severe illness	% Mortality	Hypt and Disease severity	Hypt and ICU admission	Hypt and death	Hypt and Mechanical ventilation
1	Wu C et al^31^	R	201	Wuhan, CHINA	51(43- 60)	63.7	All Hospitalized patients	19.4	10.9	4	ARDS; Death	26.4%	21.9	Associated and predictive on Multivariate analysis	NA	No	NA
2	Argenzianno et al 8	R	1000	New York	63 (50- 75)	59.6	All Hospitalized patients	60.1	37.2	23.3	Death	NS	23.2	NA	NA	NA	NA
3	Aggarwal et al 42	R	16	Des Moines, USA	75	65.5	All Hospitalized patients	56.3	31	38	Composite ICU admission, shock, or death	50	18.75	Hypertension not associated with Composite end-point			
4	Suleyman et al^41^,	R	463	Detroit, USA	57.5 (16.8)	44.1	All Hospitalized patients	63.7	38.4	23.3	ICU admission/ Mechanical ventilation /Death	39.7	20	NA	Associated. but not significant on Multivariate analysis	NA	Associated . but not significant on Multivariate analysis
5	Guan & Ni , et al 25,	R	1099	China	47(35- 58)	58.1	All Hospitalized patients	15	7.4	3.9	Composite ICU admission, Mechanical Ventilation / Death	15.74	1.4	Hypertension associated with the composite end-point. No multivariate analysis done			
6	Buckner et al 22	R	105	Washington, USA	69 (23- 97)	50	All Hospitalized patients	59	33	38	Composite : ICU admission and Death	49	33	Hypertension NOT associated with a composite end-point of ICU admission or death			
7	Chen et al^27^,	R	145	Taizhou, China	47.5 (14.6)	54.5	All Hospitalized patients	15.2	9.7	NS	Disease Severity / Death	29.7	NS	NO	NA	NA	NA
8	Guan & Liang et al 44	R	1590	China	48.9	57.3	All Hospitalized patients	16.9	9.2	5.6	Composite: ICU Admission, mechanical Ventilation, Death	16	3.1	Hypertension associated with and independently predictive of Composite end point (HR=1.58)			
9	Garg et al 23	R	1482	USA (14 STATES)	Not stated	54.4	All Hospitalized patients	12	28.3	27.8	NS	NS	NS	Outcomes not studied			
10	Huang et al 20	P	41	Wuhan, China	49(41- 58)	73	All Hospitalized patients	15	20	15	ICU Admission	32	15	NA	Not associated	NA	NA
11	Itelman et al^9^	R	162	Israel	52(20)	65	All Hospitalized patients	30.2	18.5		Death	14.8	3.1	Associated but no multivariate analysis done	NA	NA	NA
12	Li R et al^28^	R	225	CHINA	50+14	53.3	All Hospitalized patients	20.89	NR	NR	Death	16.44	0.89	Associated but no Regression done	NA	NA	NA
13	Li X et al 29	R	548	Wuhan China	60(48- 69)	50.9	All Hospitalized patients	30.3L	15.1	6.2	Disease Severity/ Mortality	49.1	32;.5	Associated and Predictive on multivari ate analysis (OR=2)	NA	NA	NA
14	Lian et al 10	R	465	Zhejiang , China	median 45 (5–88 range)	52.3	All Hospitalized patients	17.63	6.02	3	Disease Severity	10.54		Not associated	NA	NA	NA
15	Nikpourag hdam et al^24^	R	2968	Iran	50–60	65.9	All Hospitalized patients	1.99	3.81	1.25	Death	NS	8.06%	NA	NA	No (Rather, having any comorb)	NA
16	Richardson et al 21	P	5700	New York	53(52- 75)	60.3	All Hospitalized patients	56.6	33.8	18	ICU care, Renal replacement, Death	14.2	17.3	NA	NA	NA	Not associated
17	Zhang YT et al^48^	R	1350	Guangdong China	44.1±17.9	49.2	All Hospitalised patients	9.4	3.9	3.5	Disease Severity and Death	16.4	0.6	Associated (aHR=1.49)	Not assessed	Not assessed	NA
18	Wan et al^47^	R	135	Chongqing, China	47(33- 55)	53.3	All Hospitalized patients	9.6	8.9	5.2	Disease Severity/ Ventilation/ Death	29.6	2.8	Associated but no multivariate analysis done	NA	NA	NA
19	Wang D et al^2^	R	138	Wuhan, China	56(46- 68)	54.3	All Hospitalized patients	31.2	10.1	14.5	ICU Care	26.1 (ICU)	NA	NA	Associated. No multivariate analysis done	NA	NA
20	Guo^7^ et al	P	187	Wuhan, China	58.5(14.66)	48.7	All Hospitalized patients	32.6	15	15.7	Myocardial injury, Disease Severity/Death	NS	23	NA	NA	NA	NA
21	Yao et al 32	P	108	Huanggang, China	52(37- 58)	39.8	All Hospitalized patients	14.8	4.6	3.7	Disease Severity, Death	23.1	11.1	Associated but not predictive	NA	NA	NA
22	Zhang JJ et al^33^	P	140	Wuhan, China	57(25- 87)	50.7	All Hospitalized patients	30	12.1	5	Disease Severity	41.4	NS	No Association	NA	NA	NA
23	Zhao et al^34^	P	91	Hubei Province (Non- Wuhan)	46	53.8	All Hospitalized patients	20	2.2	NR	Disease Severity	33.3	2.2	No Association	NA	NA	NA
24	Zheng F et al^35^	P	161	Hunan, china	45 (33.5- 57))	49.7	All Hospitalized patients	13.7	4.3	5	Disease severity	18.6		Association, No Multivariate analysis done	NA	NA	NA
25	Zheng Y et al^36^	P	73	Shiyan China	43	54.8	All Hospitalized patients	12.3	5.5	4.1	Disease Severity	41.1	NS	NA			
26	Zhou et al^37^	R	191	Wuhan, China	56 (46- 67)	62	All Hospitalized patients	30	19	8	Disease Severity /Death	63	28.3	Not Assessed	NA	Associated but Not predictive on multivarite Analysis	NA
27	Wu & McGoogan 38	R	72314	China	NR	NR	All Diagnosed pts	NR	NR	NR	Death	NS	2.3	NA	NA	Associated. Multivaria te analysis not done	NA
28	Cummings et al^14^	P	257	New york	62 (51- 72)	67	Critically ill patients	63	36	19	Death	ALL	39%	NA	NA	Associated in Univariate but Not significant. on multivariate analysis	NA
29	Piva et al^13^	R	33	Brescia, Italy	64 (59- 72)	90.9	Critically ill patients	45	6	9.1	Death	NS	3%	NA	NA	na	NA
30	Grasselli et al^11^	R	1590	Lombardy, Italy	63(56- 70)	82	ICU Patients	49	17	21	Death		26	NA	NA	No	NA
31	Pfeffer et al^12^	R	1591	Italy	63(56- 70)	82	ICU Patients	49	17	21	Death	ALL	26%	NA	Associated No regression	NA	NA
32	Wang Y et al^26^	R	344	Tongji, China	64(52- 72)	52	ICU Patients	41	18.6	11.6	Death	ALL	38.7	NA	NA	Associated. No multivariate analysis done	NA
33	Zangrillo et al^15^	R	73	Milan, Italy	61 (54- 69)	83.6	Mechanically ventilated patients	52.9	13.6	NS	Death	ALL	23.3	NA	NA	Associated	NA

P- Prospective study, R- Retrospective study, NA- Not assessed, NS- Not stated, Hypt- Systemic hypertension, DM – Diabetes mellitus, CVD- Cardiovascular disease, ICU – Intensive care unit, ARDS- Adult Respiratory Distress Syndrome, OR- Odds ratio, aHR-Adjusted Hazard Ratio

Majority of the studies reviewed were carried out in China, seven were from centres in Wuhan, while 12 were from other centres outside Wuhan (with two being large multi-centre studies), eight studies were from the USA, four from Italy, one each from Israel and Iran. Because the COVID-19 pandemic evolved very rapidly, most of the early reports from most countries were retrospective studies. In our review, only the study by Cummings et al^14^, Huang et al^20^ and Richardson et al 21 were prospective studies. All other studies were retrospective

## Discussion

### Prevalence of Hypertension among COVID-19 patients

Observations of COVID-19 affected hospitalized individuals revealed the preponderance of hypertension as the single commonest comorbidity among COVID-19 patients in all the studies reviewed except the studies in USA by Buckner et al^22^, Garg et al^23^ and observations in Iran by Nikpouraghdam et al^24^ as well as Huang et al20 in China where diabetes mellitus was the most prevalent co-morbidity. Generally speaking, the prevalence of hypertension was lower in hospitalised COVID-19 patients in China than in Europe or USA. It ranged between 7.2%–32.6% in Chinese cohorts, 2, 7, 25–38 it was 30.2% in Israeli patients9 while in European patients it ranged from 45 to 52.9%^12^, ^15^, ^39^, ^40^ and it was12% to 63.7% in the USA.^8^, ^14^, ^22^, ^23^, ^41^, ^42^

The high prevalence of hypertension among hospitalised patients was initially thought to be linked to the advanced age and the predominance of the male gender in hospitalized COVID-19 patients. Pirola et al^39^ in a meta-regression of the meta-analysis by Zheng Z et al^43^ submitted that age and not sex accounted for the high prevalence of hypertension among other co-morbidities in COVID-19 patients who had critical illness or who died. However among the relatively young cohorts of Guan et al^44^, Chen et al^27^, Suleyman et al^41^, Yao et al^32^, Zheng F et al^35^, Zheng Y et al^36^ with mean age less than 50 years, hypertension was also the most prevalent comorbidity. Suleyman et al studied a cohort with a mean age of 44 years with a prevalence of hypertension of 63.7%.

It is also worthwhile to consider that in some cases, the prevalence of hypertension in cohorts of COVID-19 patients reflected the fact that hypertension is the commonest comorbid condition in the general population. In a large Chinese COVID-19 combined in-patient and outpatient database of 20,982 patients, the proportion of self-reported hypertension was 12.6%, which is similar to the population prevalence data of 10.9% in China for self-reported hypertension.^45^

The association between hypertension and hospitalization rates for COVID-19 may also be related to the fact that hypertension is a proxy for the presence of other cardiovascular risk factors such as diabetes, coronary artery disease and cerebrovascular disease.^16^ Hence, the observed association between hypertension and COVID-19-related hospitalizations is thought to be confounded by the presence of other comorbidities.^16^

### Hypertension and COVID-19 disease severity

Though all the works in this review did not assess the relationship between hypertension and COVID-19 disease severity, this relationship was explored by a majority of workers. However, it was observed that there is no uniform severity scale across all the studies. For example Li X, et al 29, Yao et al,^32^ and Guan et al^44^ defined severe COVID-19 according to the 2019 clinical practice guideline of the American Thoracic Society and the Infectious Diseases Society of America for the diagnosis and treatment of adults with community acquired pneumonia.^46^ Lian et al 10 based their own classification of severity on the 5th edition of the Chinese diagnosis and treatment scheme for SARS-Cov-2 while Wang Y et al^26^ and Wu & McGoogan^38^ used the 6th edition which identified severe illness as that which was characterized by dyspnea, respiratory rate ≥30/minute, blood oxygen saturation ≤93%, PaO2/FiO2 ratio <300, and/or lung infiltrates >50% within 24–48 hours. Wan et al^47^ also used criteria similar to the Chinese algorithm. Overall, however, the criteria for defining severe disease in both guidelines are similar except that the presence of septic shock or respiratory failure requiring mechanical ventilation which were additional indices of severe disease according to the American Thoracic Society guideline were further classified into an additional group named critical disease in the Chinese diagnosis and treatment scheme for SARS-Cov-2. For the purpose of this review patients with severe and critical disease were regarded as having ‘severe’ disease. Other classification schemes used by Itelman et al^9^ and Zhang et al^48^ though not as structured as either of the foregoing however captured similar features in their description of severe disease. Zheng Y et al^36^ did not however state the details of their classification of disease severity.

The lowest prevalence of severe disease COVID-19 patients was 15.7% reported by Guan and Ni et al^25^ in a Chinese cohort while the highest prevalence of severe disease was 63% by Zhou et al/span>37 also from China. The median age of Zhou et al's patients was 56years while Guan and Ni et al's patients had a median age of 47 years suggesting that age may have influenced the occurrence of severe disease.

While Chen et al 27, Zhao et al^49^ and Zhou et al^37^, Zhang JJ et al^33^ found no association between hypertension and disease severity, Aggarwal et al^42^ found an association between hypertension and a composite end point of disease severity, need for ICU care and mortality. Several other workers who found an association between hypertension and disease severity but either did not subject this to multivariate analysis or found hypertension not to be predictive of severe disease after multivariate analysis include, Itelman et al^9^, Li R et al^28^, Yao et al^32^, and Zheng F et al^35^. Li X et al^29^, Wu et al^31^, Zhang Y et al^48^ and Guan & Liang et al^50^, demonstrated that hypertension was not only associated with but also independently predictive of developing severe COVID-19 disease. Overall majority of studies reviewed either did not show any association between hypertension and disease severity, or showed an association which was not significant on multivariate analysis. Only 3 studies demonstrated that hypertension independently predicted severity of disease.

For studies that didn't show hypertension as being independently predictive of disease severity, despite observed association on univariate analysis, the observed association may have been confounded by patient's age and preponderance of male gender in most patient cohorts. Conversely, explanations for the significant predictive relationship of hypertension with development of severe COVID-19 disease on multivariate analysis may be related to immune mechanisms, immune dysregulation and inflammation underlying the pathogenesis of hypertension and the mediation of target organ damage in established hypertension.

It has been suggested that pro-inflammatory immune mechanisms play an important role in the pathogenesis of hypertension. This is supported by the demonstration of increased levels of circulating IgG in the serum of hypertensives.^51^, ^52^ Increased numbers of central memory CD8+ T cells, activated CD8+ T cells producing interferon gamma (IFNγ) and tumour necrosis factor (TNF), TH17 cells53 interleukin (IL)-2, IL-6, and IL-754 have also been reported in patients with hypertension. The association of these cytokines with the development of hypertension has been demonstrated in experimental 55 clinical observational^53^, as well as in interventional studies. 54 Noteworthy is the fact that these immune responses can induce kidney injury and also interfere with sodium excretion, further contributing to the elevation of blood pressure.^56^

An increase in systemic IL-2, IL-6, and IL-7, granulocyte colony-stimulating factor, C-X-C motif chemokine 10 (CXCL10), chemokine (C-Cmotif) ligand 2 (CCL2), and tumour necrosis factor-α (TNF- α) has been observed in patients with COVID-19.20 Rapid deterioration in COVID-19 patients is associated with a pro-inflammatory cytokine storm. Some of the key mediators of the cytokine storm are these inflammatory mediators which are also elevated in and associated with regulating immune-inflammatory responses in hypertension. 57 However only Suleyman et al subjected their observation to multivariate analysis and found no relationship after controlling for confounders. Other workers such as Huang C et al^20^ found no significant association between hypertension and need for ICU care. However, their study is limited by a small sample size of 41. The complete picture of the relationship between hypertension and need for ICU care is hampered by the quality of studies which mostly carried out univariate analysis.

### Hypertension and the need for mechanical ventilation

Among the studies in our review, only Suleyman et al^41^ evaluated the relationship between hypertension and need for mechanical ventilation as a single primary outcome. Suleyman et al found a significant association. All the other studies assessing this relationship related hypertension to a composite outcome measure comprising admission to ICU, mechanical ventilation and death. This composite outcome measure was adopted in the studies by Guan & Ni et al^25^ and Guan & Liang et al^44^ because all the individual components were reportedly the prominent outcomes of the earlier H7N9 infection experienced in China and South Asia. 25, 44 Guan & Ni et al^25^ found that hypertension was associated with this composite endpoint but did not carry out multivariate analysis. In another study by Guan & Liang et al however, hypertension with an odds ratio of 1.58 was independently predictive of the composite outcome and this was after adjusting for age and smoking status.^44^

The relationship between hypertension and the need for mechanical ventilation may be related to many factors including but not limited to the higher frequency of clustering of cardiovascular risk factors with attendant exaggerated inflammatory response among study participants. The increased transpulmonary pressure required for mechanical ventilation may also further alter the course of the disease negatively and that may explain the rationale for the combined effect of hypertension on the three variables studied by Guan & Liang et al.

### Hypertension and the risk of death.

The high representation of hypertension among comorbid risk factors of COVID-19 patients has implications for the relationship between hypertension and mortality. Grasseli et al^11^, Cummings et al^14^, Zangrillo et al^15^, Wang Y et al^26^, Zhou et al^37^, Wu & McGoogan^60^ found that hypertension was associated with mortality in COVID-19 patients. However, only Zangrillo et al Zhou et al and Cummings et al subjected the association to multivariate analysis with different results. While Zangrillo et al found hypertension to be an independent predictor of mortality (Odds Ratio 6.15), Cummings et al and Zhou et al found no such relationship. However, Zangrillo et al recruited only COVID-19 patients who were mechanically ventilated hence more ill than all the other cohorts which comprised all hospitalized patients with different levels of disease severity. The observation by Wu & McGoogan is also worthy of note because they studied the largest single cohort in our review comprising 72,314 patients. It has been suggested that it is not the presence of hypertension only contributes to the increased mortality but the fact that hypertensives frequently have more underlying health problems than others. Data has confirmed that patients with multiple comorbidities are likely to fare worse than those with single comorbid conditions. 32

Other workers who found no association between hypertension and mortality include Li X et al^29^, Wu C et al^31^, Yao et al^32^ and Nikpouraghdam et al^24^. Patient demographic parameters such as age and gender distribution and the different patterns of comorbidities in the different patient cohorts can explain the different observations of the effect of hypertension on mortality among patients with COVID-19.

A few studies rather than study the effect of hypertension on individual outcomes studied its effect on a composite outcome of disease severity, need for ICU care and death. Aggarwal et al 42 found no association between hypertension and a composite end point. However, this study being one of the early reports from the USA is limited by a small sample size of 16. A larger study of 1590 patients by Guan & Liang et al^44^ however found an association and confirmed that hypertension independently predicted a composite end-point. Buckner et al^22^ however found no association between hypertension and a composite endpoint of ICU care and death.

The presence of an association between hypertension and death may be accounted for by the effect of immune dysregulation described earlier. It has been described that those processes not only underly the development of hypertension but also contribute to target organ damage. The additive effect of pre-existing cytokine activation and that which is triggered by SARS-Cov-2 may be what predisposes to mortality.

Another reason for the association of hypertension with COVID-19 patient mortality may be a synergy of hypertension with myocardial injury and other effects of SARS-Cov-2 on the cardiovascular system which are not frequently assessed. Guo et al^7^ showed that myocardial injury (assessed using troponin T) occurred frequently among hospitalized COVID-19 patients and hypertensive COVID-19 patients with myocardial injury accounted for the sub-group of patients with the highest mortality.

The unfolding new information about the relationship between cardiovascular risk factors including hypertension and composite outcomes such as mortality and need for ICU care will be further understood in the future as more information accrues to the scientific world especially through longitudinal reviews.

### Limitations

A main limitation of most of the studies reviewed was that self-reporting of hypertension and indeed other comorbidities on admission was used. Under-reporting of comorbidities, stemming from the lack of awareness and/or the lack of diagnostic testing, may contribute to the underestimation of the true strength of association with the clinical prognosis. Under-reporting of comorbidities could also lead to over-estimation of the strength of association with adverse outcomes.

In a few of the studies too, some patients, though having met study outcome objectives were still on hospital admission at the time of publication and may have evolving outcomes different from what has been published. However, because the COVID-19 disease is a rapidly evolving public health issue, every stage of data captured will still reflect a part of the complete picture of the disease.

## Conclusion

Hypertension is the commonest co-morbidity in hospitalised COVID-19 patients. It is frequently associated with development of severe disease, need for ICU care, need for mechanical ventilation and death. However, the strength of evidence for this relationship is weak as only few studies rigorously control for confounders. In many study cohorts this association may have been influenced by age and gender and presence of other comorbidities. The role of myocardial injury and other effects of the virus on the cardiovascular system in potentiating the effect of hypertension on mortality also requires further study. Since the pandemic is still escalating, well-planned prospective studies are needed to properly define the relationship between hypertension and clinical outcomes.
